# A dataset for internet of things based fish farm monitoring and notification system

**DOI:** 10.1016/j.dib.2020.106457

**Published:** 2020-10-24

**Authors:** Arif Istiaq Arafat, Tasmima Akter, Md. Ferdous Ahammed, Md. Younus Ali, Abdullah-Al Nahid

**Affiliations:** Electronics and Communication Engineering Discipline, Khulna University, Khulna-9208, Bangladesh

**Keywords:** Water quality factors, Fish farm monitoring, Real time monitoring, Digital sensors, Machine learning, Internet of things, Micro-controller, Notification system

## Abstract

Water quality depends on many factors. Some of them are essential for maintaining the minimum sustainability of water. Because of the great dependence of fishes on the condition of the aquatic environment, the water quality can directly affect their activity. Therefore monitoring water quality is a very important issue to consider, especially in the fish farming industry. In this paper a digital fish farm monitoring system is introduced and a collection of experimental data of water quality monitoring was presented, which were directly collected from a fish pond. As the quality factor of water affects its aquatic life form sustainability, therefore the quality factors of the water were measured using digital sensors. Temperature, pH factor and Turbidity were selected as the basic quality factors to measure. The dataset contains data recorded from two different water levels to analyze the aquatic environment more efficiently. Each level has 9623 sets of data of the selected parameters. Collection was continued all day long for several days. Later collected sensor data were analyzed as short period time series to find its properties. Machine Learning regression method was used to predict near future conditions. Moreover data were processed to find any repetitive patterns in its properties. This dataset represents the exact condition of the environment of the fish pond. Therefore it can be used to develop a system to monitor fish farms digitally. Using these data in machine learning, predicting the future is possible for advance monitoring of a fish farm. The dataset is available in Mendeley Data[1].

## Specifications Table

**Table:** 

Subject	Engineering
Specific subject area	Application of Electronics Engineering, Internet of Things (Iot) and Computer Network in monitoring the sustainability, quality and condition of the aquatic environment of a fish farm.
Type of data	Ms Excel Table Graphs
How data were acquired	Using three types of digital sensor data were recorded directly from the natural environment. Temperature, pH and Turbidity. Later these data were uploaded in a cloud database using ESP8266 Wi-Fi Module. At the same time they were also stored in an electronic storage device. Monitoring all the digital sensors and storing process was done by an Arduino Mega microcontroller board. After that data were analyzed and graphically represented by Matlab.
Data format	Raw Analyzed Labelled
Parameters for data collection	Natural aquatic environment was the venue for data collection, at natural temperature and lighting. Recorded for 24 hours a day. Data was recorded automatically from environment without any interruption.
Description of data collection	A fish pond was the venue for collecting data, where two sets of waterproof digital sensors were used in this collection. Sensors were immersed in the water and collected data at the same time. Arduino Mega as the processing unit recorded and stored all these sensors’ data. Data were recorded for 24 hours a day, from 15 January 2020 to 22 January 2020. After collection data was uploaded to a cloud database using the Internet of Things (IoT) and an electronic storage device stored these data also. Data were represented in a tabular format and graphically afterwards.
Data source location	Institution: Khulna University City/Region: Khulna Country: Bangladesh Latitude and longitude: 22.802°N, 89.533°E
Data accessibility	Repository name: Mendeley Data Data identification number: http://dx.doi.org/10.17632/34rczh25kc.4 Direct URL to data: https://data.mendeley.com/datasets/34rczh25kc/4

## Value of the Data

• This dataset can be used for analyzing the condition of water in any fish farm to find its sustainability. Moreover, training machine learning regression method with it will help us to forecast the aquatic environment in the near future. Also any anomaly can be detected very quickly in the water quality factors by using the machine learning process. Automatic fish farm monitoring will be possible with it [2].
• This dataset will be beneficial for the fish farming industry. It will be also beneficial for environmental scientists as it contains raw natural data.
• Further these data can also be used for analyzing local geographical characteristics and discover new scopes of farming.
• If such a data collection system is to be implemented in every fish farm throughout the country, a central monitoring and data base system can be built. It will help to compute the overall fish production in the country and help to make statistics of national profit, net productions, laggings, type of productions, possible productions, lag of any productions and many more.

## 1 Data Description

**Fig. 1:**
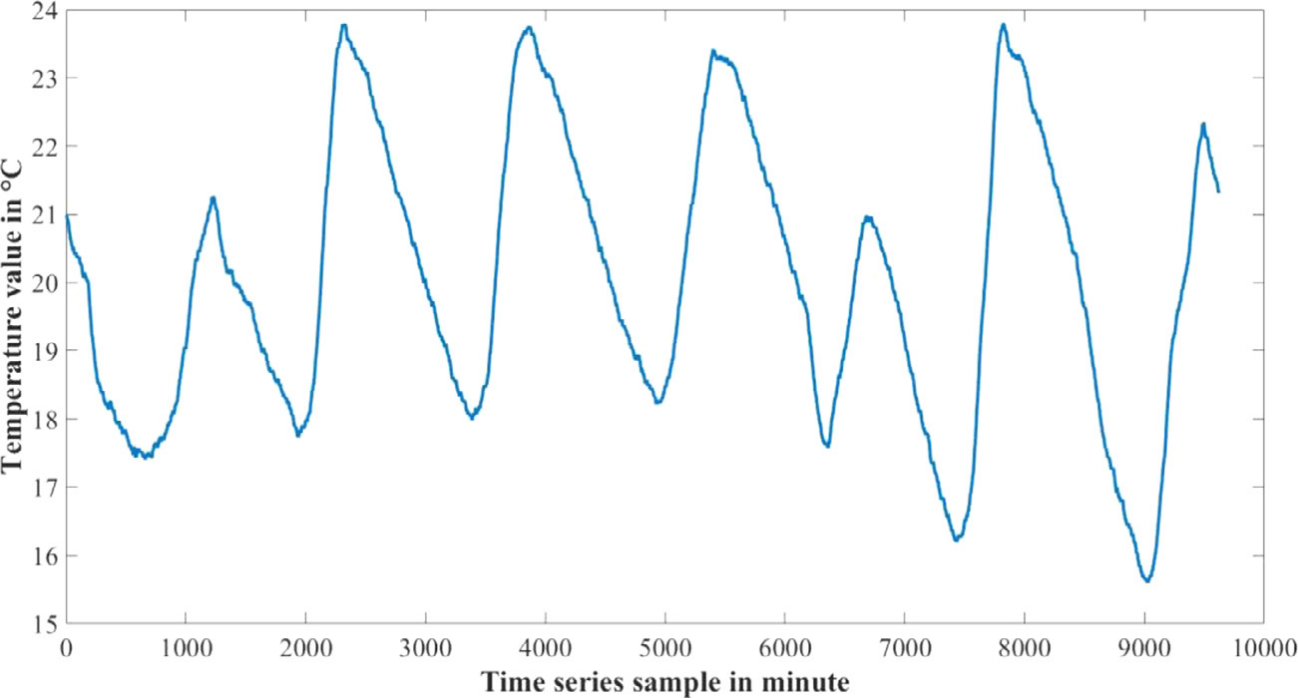
Temperature data from 30 cm underwater.

**Fig. 2:**
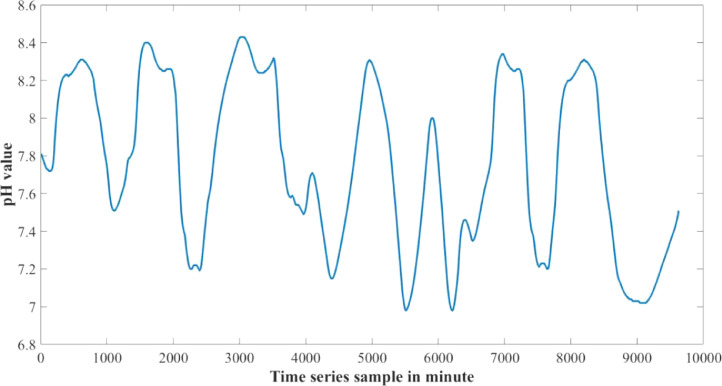
pH data from 30 cm underwater.

**Fig. 3:**
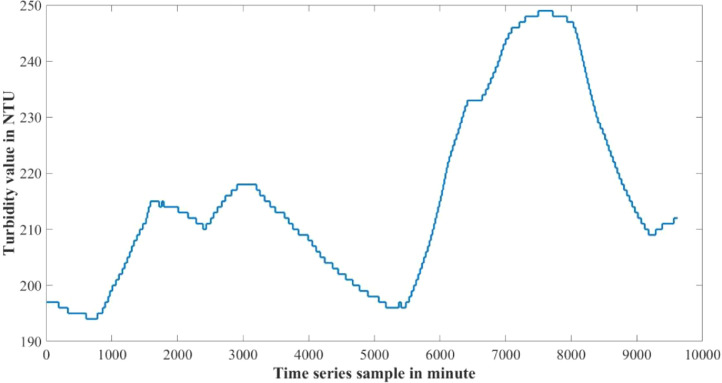
Turbidity data from 30 cm underwater.

**Fig. 4:**
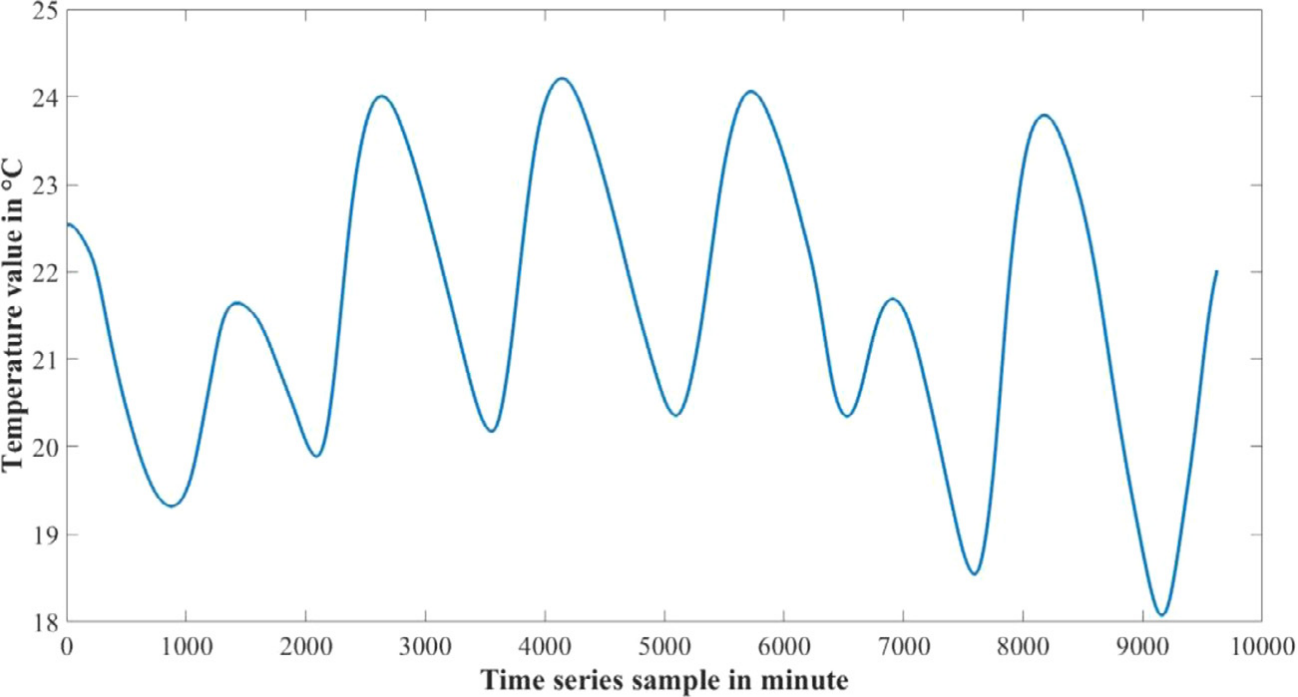
Temperature data from 60 cm underwater.

**Fig. 5:**
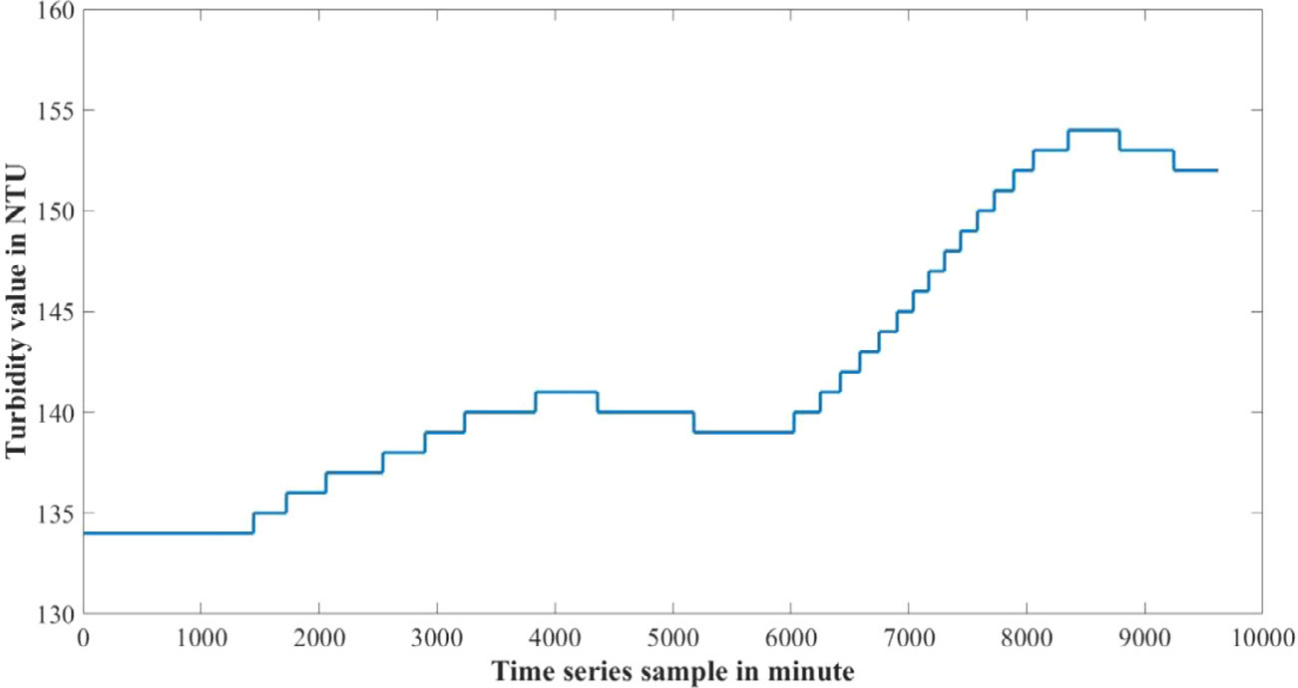
Turbidity data from 60 cm underwater.

The dataset presented in this article is available in the Mendeley data repository [1]. The dataset has two data files which represents relative information from two different depths of water level. Table 1 and Table 2 show sample data of the dataset. The “Sensor data for 30 cm.xlsx” file includes Temperature sensor data, pH sensor data and Turbidity sensor data from 30 cm below the water surface. It has 9623 sets of data containing three data samples for each set of respective sensors. The “Sensor data for 60 cm.xlsx” file has Temperature and Turbidity sensor data from 60 cm below the water surface. It also has 9623 sets of data containing two data samples for each set of respective sensors. pH rating was not collected from 60 cm depth as changes in pH in a small area gets normalized quickly with respect to the surrounding area, so no significant changes are observed. For the both data files time samples are identical. Row-wise day and night time cycle over the experimental time period is presented in Table 3. Raw sensor data from 30 cm underwater are presented graphically in Figs. 1–3 and raw sensor data from 60 cm underwater are presented graphically in Figs. 4–5. In these graphs X-axis represents the time series samples in minutes and Y-axis represents the data values. Table 4 shows the maximum and minimum values recorded by the sensors over the experimental time period. Table 5 shows the mean value of the parameters over the experimental time period.

**Table 1:** Sample data from “Sensor data for 30 cm.xlsx” dataset.

Date and Time	Temperature (°C)	pH	Turbidity (NTU)
2020-01-15 16:00:35	20.99	7.81	197
2020-01-15 16:01:33	20.99	7.81	197
2020-01-15 16:02:33	20.98	7.81	197
2020-01-15 16:03:22	20.98	7.81	197
2020-01-15 16:04:26	20.98	7.81	197
2020-01-15 16:05:21	20.97	7.81	197
2020-01-15 16:06:20	20.97	7.81	197
2020-01-15 16:07:17	20.95	7.81	197
2020-01-15 16:08:59	20.94	7.81	197
2020-01-15 16:10:00	20.93	7.81	197
2020-01-15 16:11:11	20.92	7.81	197

**Table 2:** Sample data from “Sensor data for 60 cm.xlsx” dataset.

Date and Time	Temperature (°C)	Turbidity (NTU)
2020-01-15 16:00:35	22.54	134
2020-01-15 16:01:33	22.54	134
2020-01-15 16:02:33	22.54	134
2020-01-15 16:03:22	22.54	134
2020-01-15 16:04:26	22.54	134
2020-01-15 16:05:21	22.54	134
2020-01-15 16:06:20	22.54	134
2020-01-15 16:07:17	22.54	134
2020-01-15 16:08:59	22.54	134
2020-01-15 16:10:00	22.54	134
2020-01-15 16:11:11	22.54	134

**Table 3:** Row-wise day and night cycle from the data files.

No. of experimental day	Row-wise Day time(6:00am-6:00pm)	Row-wise Night time(6:00pm-6:00am)
1	2 (4:00pm)-116	117-810
2	811-1445	1446-2169
3	2170-2855	2856-3633
4	3634-4410	4411-5155
5	5156-5885	5886-6553
6	6554-7189	7190-7832
7	7833-8476	8477-9077
8	9078-9624 (4:25pm)	-

**Fig. 6:**
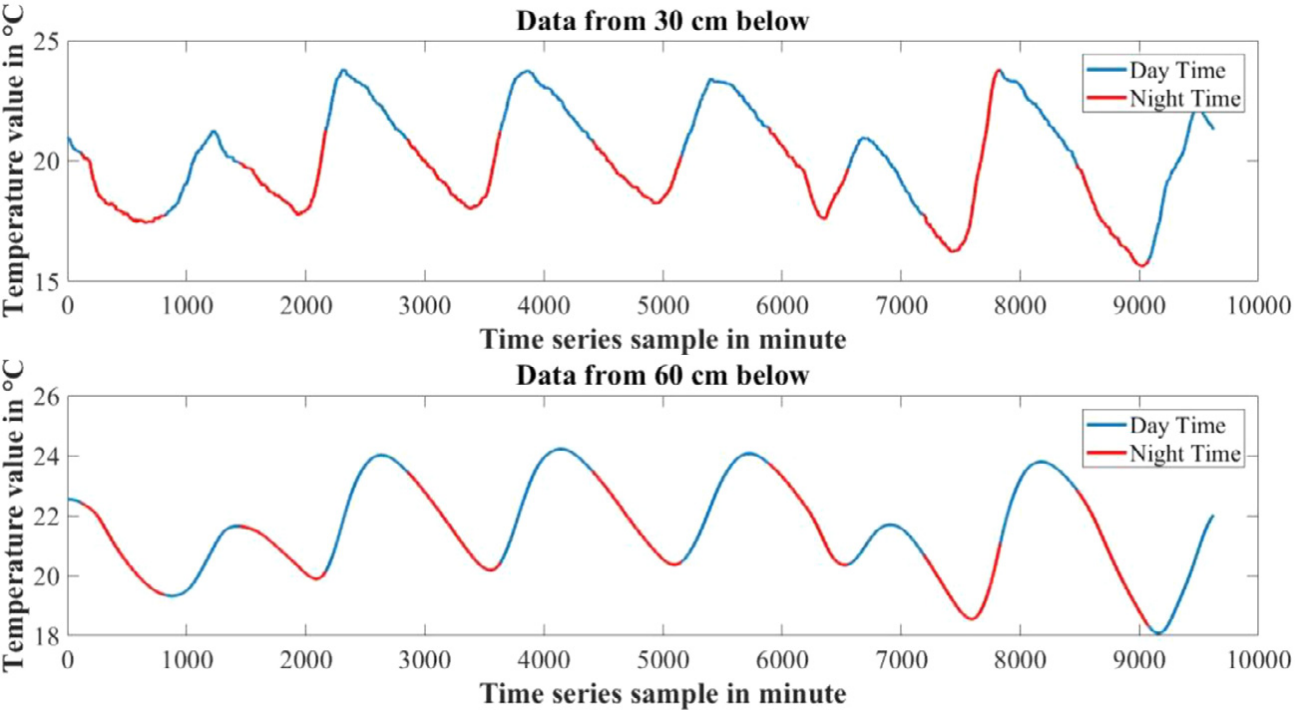
Change in Temperature in day and night time.

**Fig. 7:**
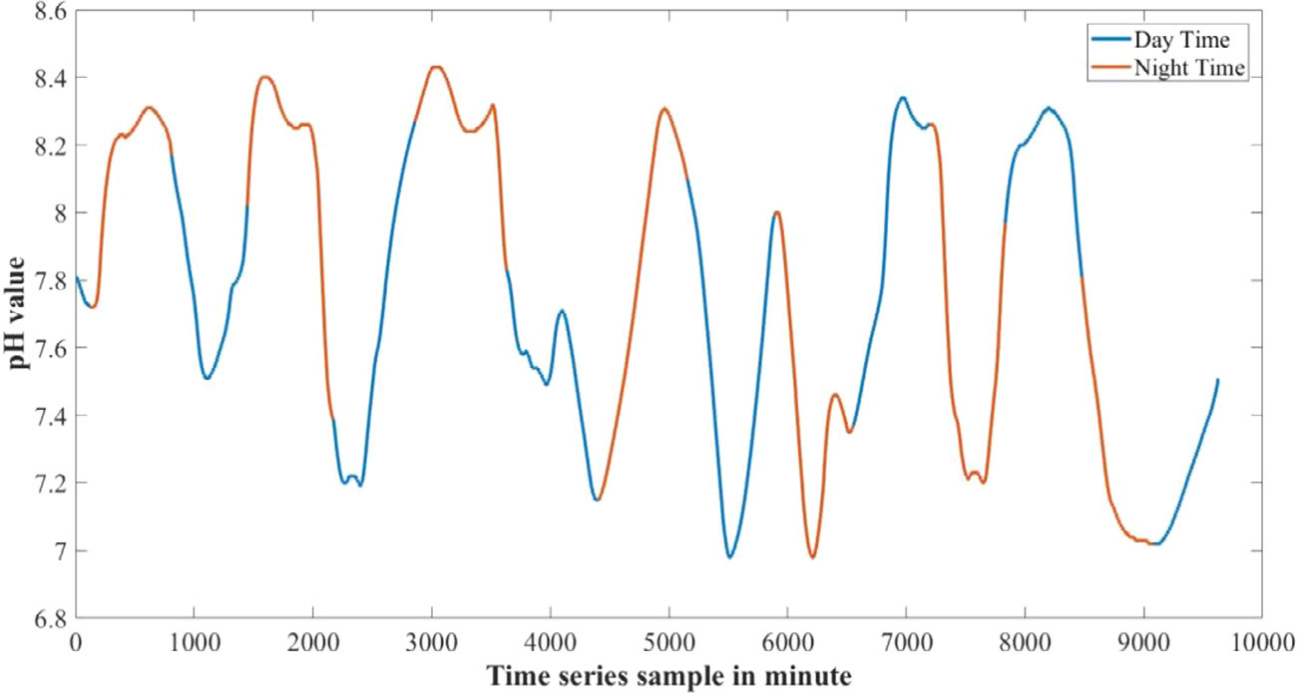
Change in pH in day and night time.

Day and night time has different levels of temperature and pH rating and changes at a different rate. Temperature increases at the day time and decreases at the night time, where most of the time pH also has a tendency to increase at day time and slowly decrease at night time. Turbidity does not affect much by the day night cycle. With the increase of water depth temperature changes at a very slower rate. As a result the effect of day and night cycle is comparatively lower in the sensors 60 cm underwater. Fig. 6, Fig. 7 show the changes in temperature and pH with respect to day and night cycle. In the graphs blue color denotes day time data from 6:00am to 6:00pm and red color denotes night time data from 6:00pm to 6:00 am. Table 6 shows the mean day and night time parameters over the experimental time period.

**Fig. 8:**
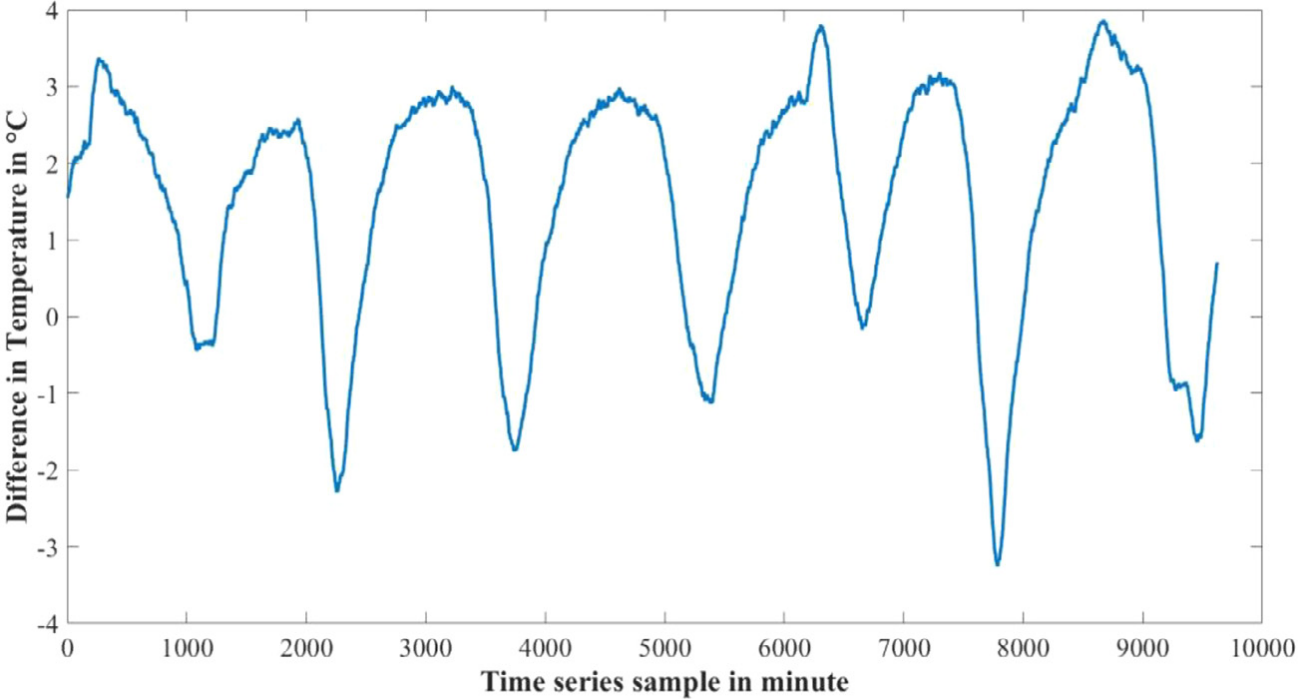
Temperature difference between 30 cm and 60 cm underwater.

**Fig. 9:**
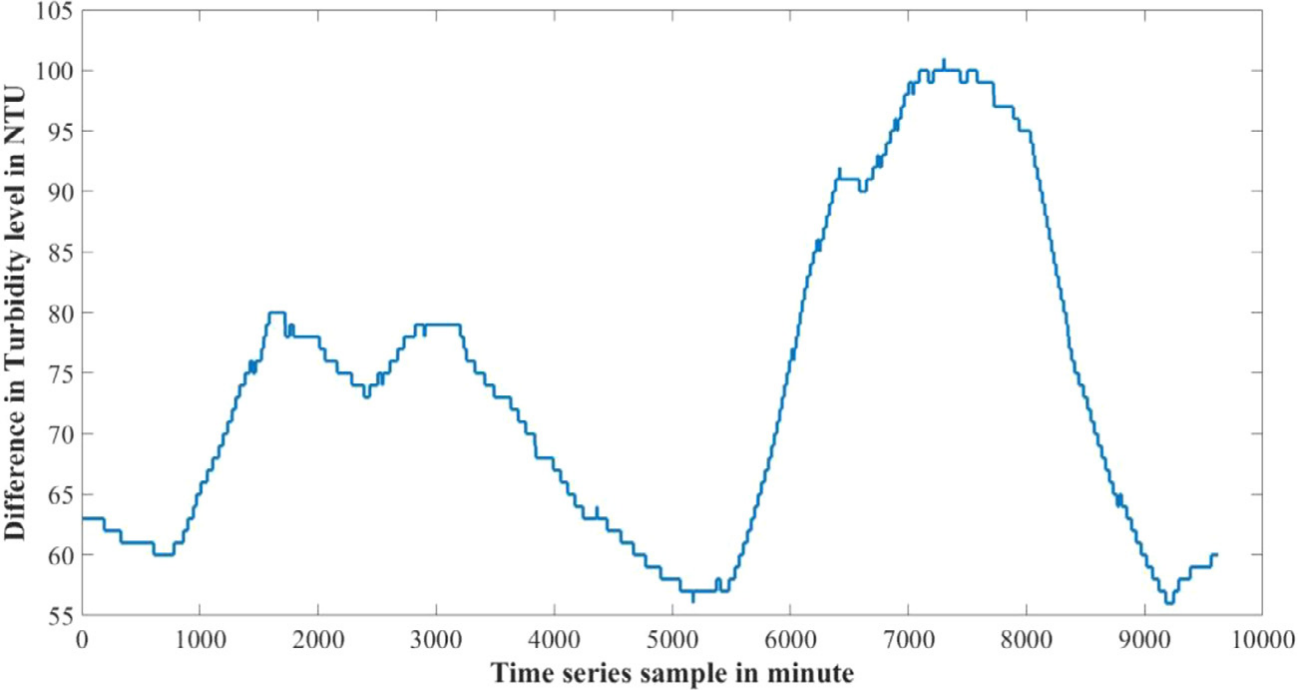
Turbidity difference between 30 cm and 60 cm underwater.

Temperature and turbidity values are not the same for both 30 cm and 60 cm levels. These two levels have difference in sensors values. Temperature increases with the depth of water level. As a result temperature rating in 60 cm underwater is warmer than 30 cm underwater. On the other hand turbidity level in 60 cm underwater is much lower than 30 cm underwater. Fig. 8, Fig. 9 show the difference of parameter values between 30 cm and 60 cm underwater.

**Fig. 10:**
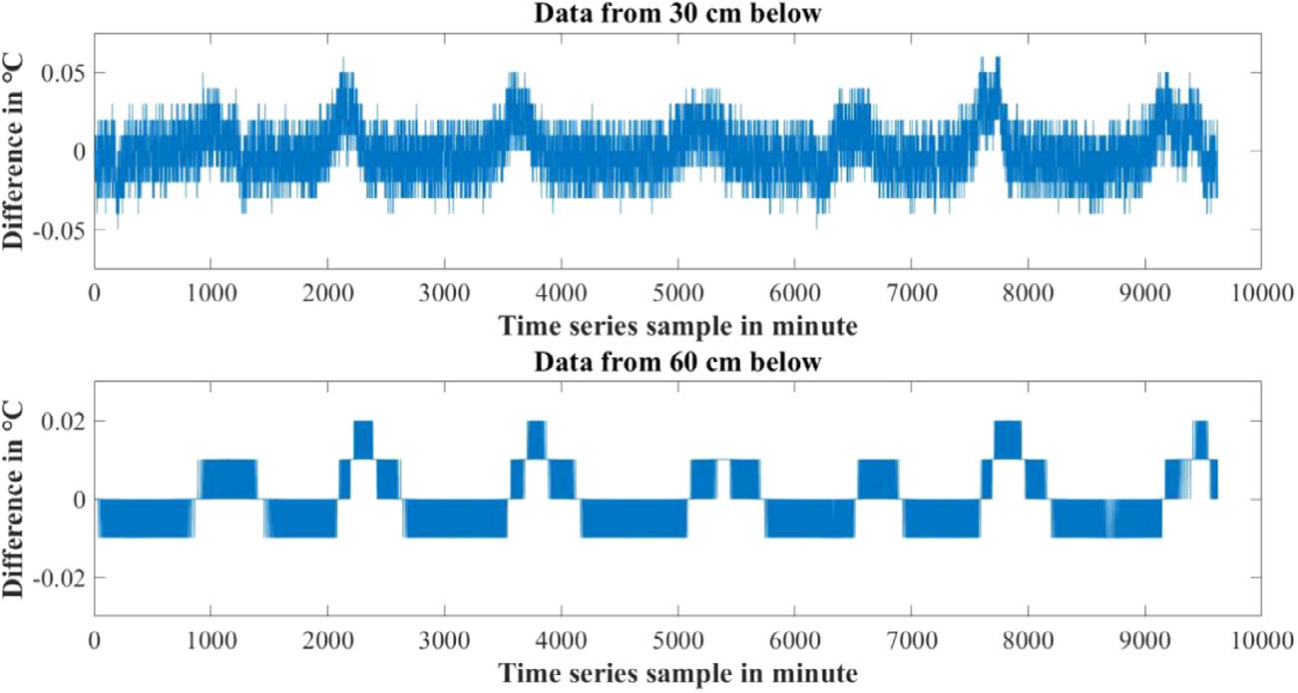
Difference between adjacent values of Temperature.

**Fig. 11:**
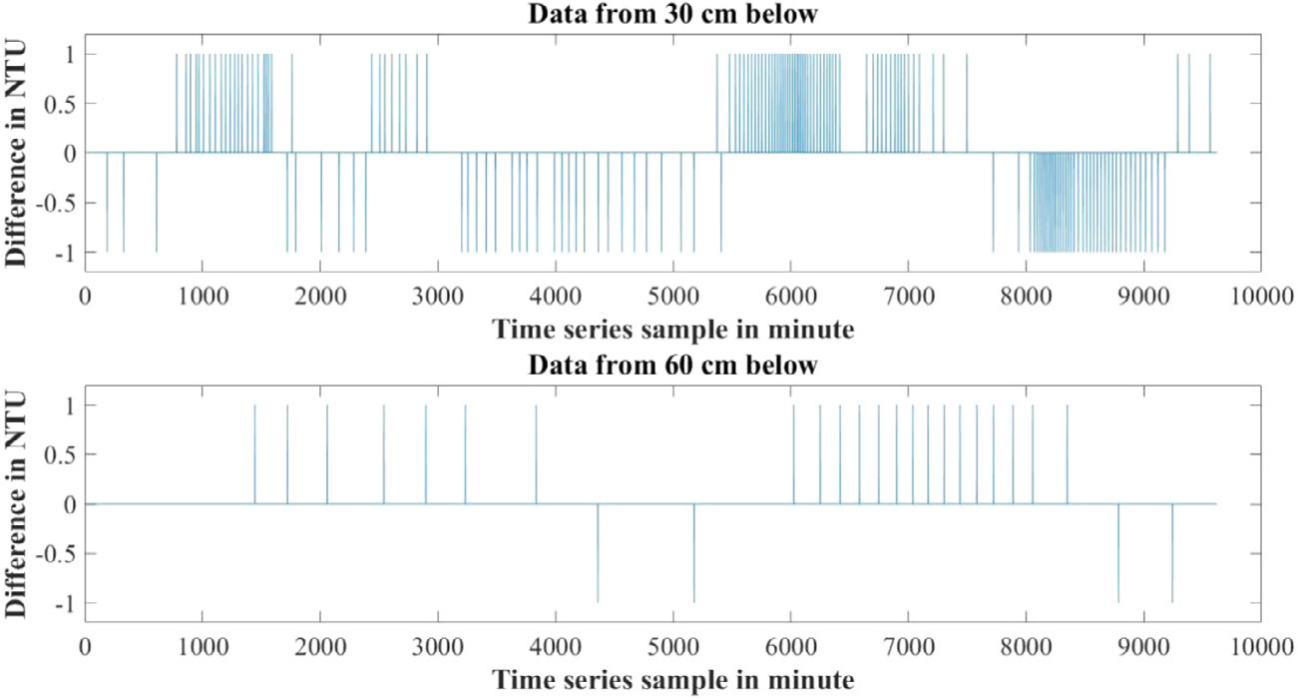
Difference between adjacent values of Turbidity.

**Fig. 12:**
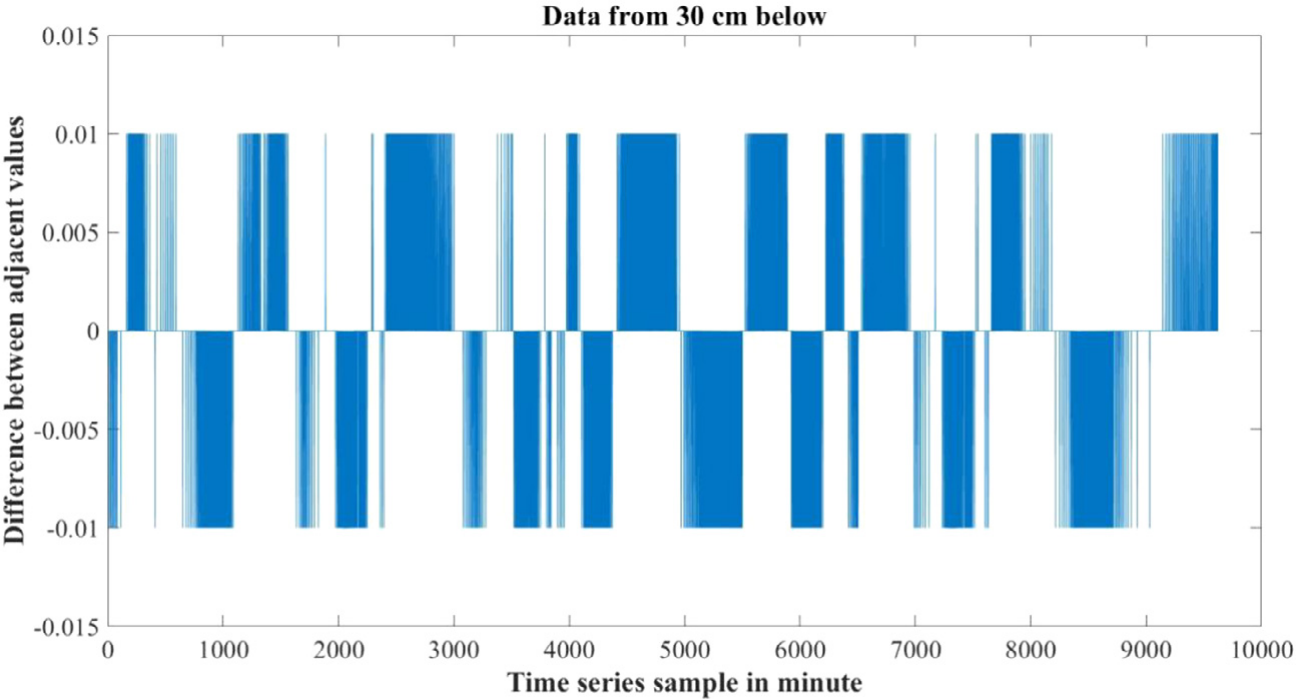
Difference between adjacent values of pH.

Furthermore the rate of change of parameters are not the same for both water levels also. Figs. 10–12 show the difference between adjacent values of each parameter for each water level depth. Near surface temperature of water level gets affected by the environmental temperature easily. As a result temperature in 30 cm underwater has a rapid changing rate and more fluctuation in the adjacent values. Temperature in 60 cm underwater does not change that rapidly. The rate of change is slow and steady. The adjacent values also show some periodic nature that repeats itself. Same properties are also found in turbidity values. Turbidity changes very slowly in 60 cm than 30 cm underwater. Yet, both level has a steady changing rate between the adjacent values. pH also has a steady changing rate between the adjacent values and changes slowly.

**Table 4:** Maximum and Minimum values recorded by the sensors over the experimental time period.

S/N	Parameter Name	Maximum value	Minimum Value	Unit
1	Temperature sensor in 30 cm depth	23.81	15.6	°C
2	Temperature sensor in 60 cm depth	24.21	18.08	°C
3	pH sensor in 30 cm depth	8.43	6.98	-
4	Turbidity sensor in 30 cm depth	249	194	NTU
5	Turbidity sensor in 60 cm depth	154	134	NTU

**Table 5:** Mean value of the parameters over the experimental time period.

S/N	Parameter Name	Value	Unit
1	Mean temperature value in 30 cm depth	20.0048	°C
2	Mean temperature value in 60 cm depth	21.5126	°C
3	Mean pH value in 30 cm depth	7.7579	-
4	Mean turbidity value in 30 cm depth	216	NTU
5	Mean turbidity value in 60 cm depth	142	NTU

**Table 6:** Mean values of day and night time parameters over the experimental time period.

S/N	Parameter Name	Day time	Night time	Unit
1	Mean temperature value in 30 cm depth	21.179	18.830	°C
2	Mean temperature value in 60 cm depth	21.979	21.047	°C
3	Mean pH value in 30 cm depth	7.688	7.828	-
4	Mean turbidity value in 30 cm depth	214	217	NTU
5	Mean turbidity value in 60 cm depth	142	141.71	NTU

**Fig. 13:**
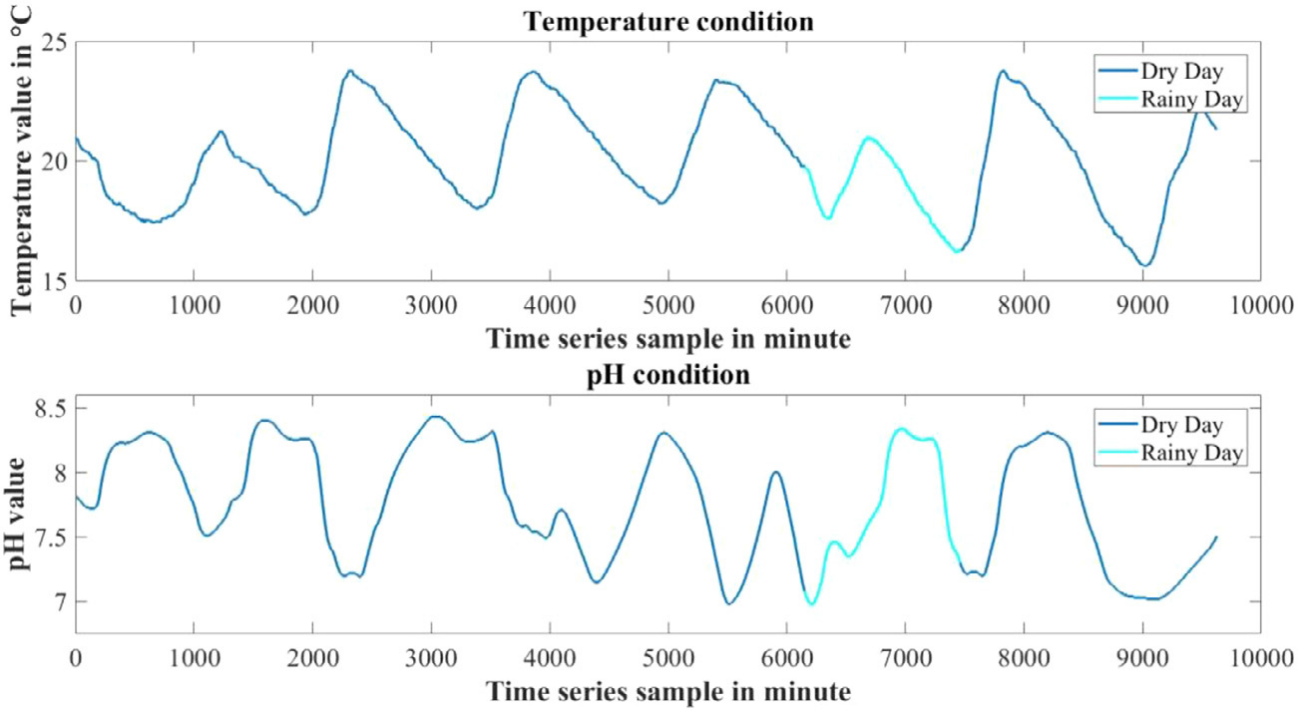
Temperature and pH data of dry day and rainy day from sensors 30 cm underwater.

**Fig. 14:**
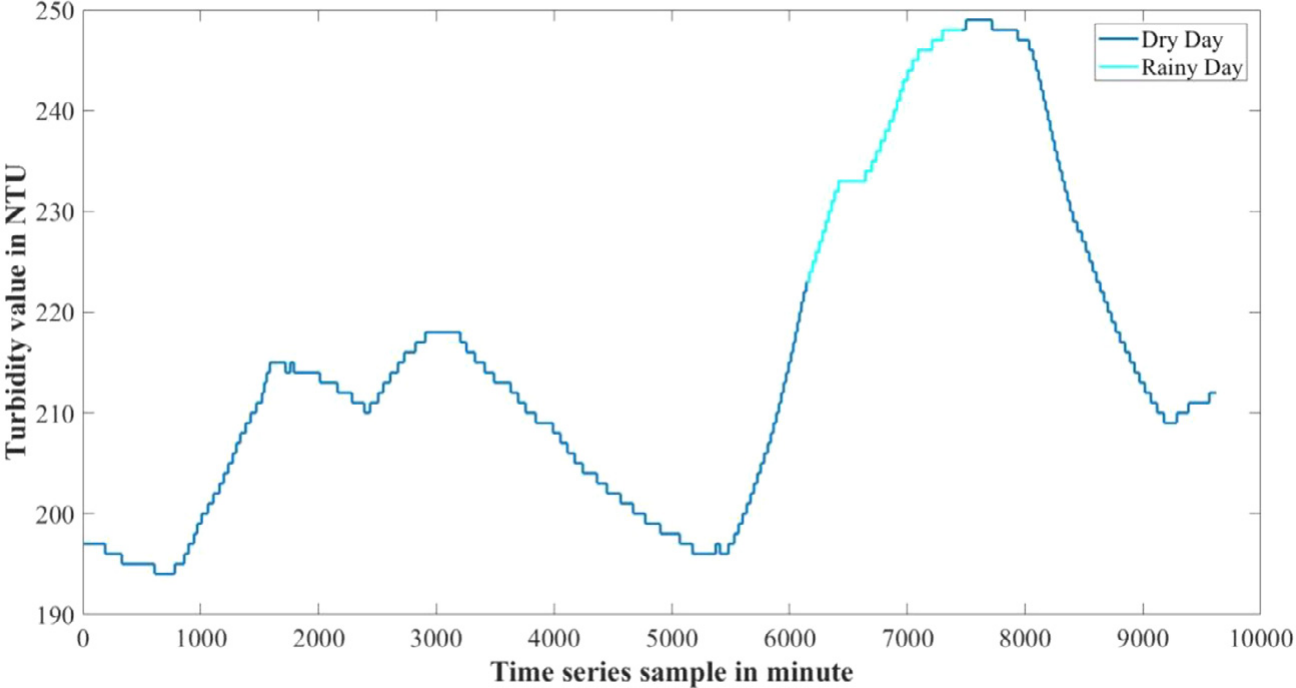
Turbidity data of dry day and rainy day from sensors 30 cm underwater.

The experiment was conducted for seven days and among them there was a rainy day. In Fig. 13, Fig. 14 data from 30 cm underwater are presented where dry day's data are represented in blue color and the rainy day's data are represented as cyan color. During this period mean value of temperature decreased and turbidity increased than the other experiment days. The mean value of the parameters of dry days are described in Table 7 and the mean value of the parameters of the rainy day are described in Table 8.

**Table 7:** Mean value of the parameters of dry days.

S/N	Parameter Name	Value	Unit
1	Temperature sensor in 30 cm depth	19.8695	°C
2	Temperature sensor in 60 cm depth	21.3775	°C
3	pH sensor in 30 cm depth	7.6878	-
4	Turbidity sensor in 30 cm depth	217	NTU
5	Turbidity sensor in 60 cm depth	145	NTU

**Table 8:** Mean value of the parameters of the rainy day.

S/N	Parameter Name	Value	Unit
1	Temperature sensor in 30 cm depth	18.7704	°C
2	Temperature sensor in 60 cm depth	20.9467	°C
3	pH sensor in 30 cm depth	7.7256	-
4	Turbidity sensor in 30 cm depth	238	NTU
5	Turbidity sensor in 60 cm depth	144	NTU

**Fig. 15:**
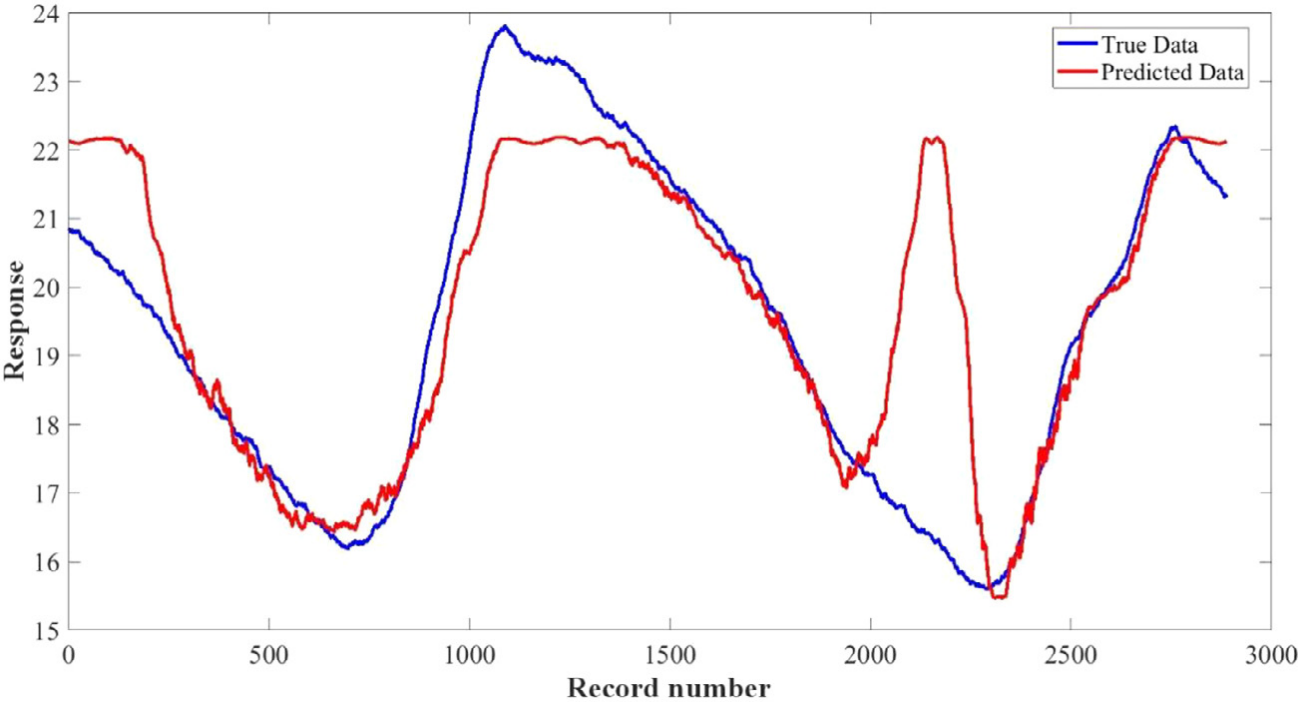
Regression method response plot of Temperature sensor 30 cm underwater.

**Fig. 16:**
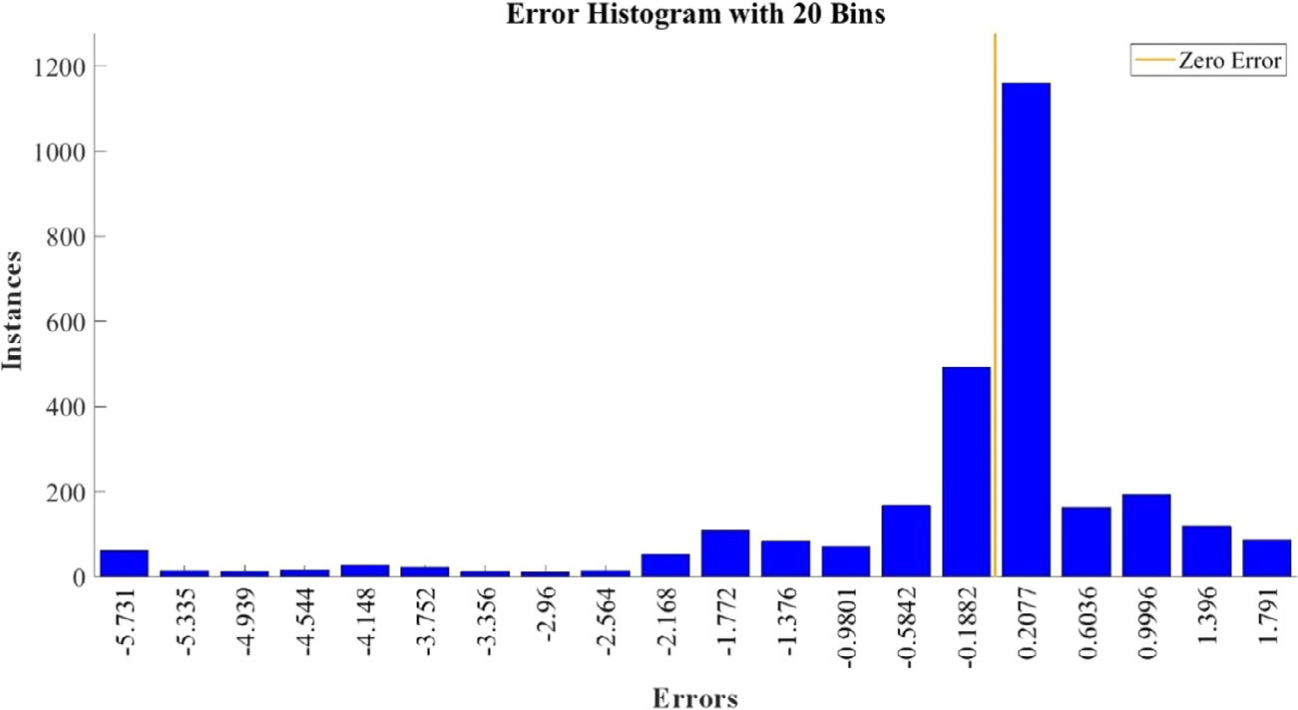
Error histogram plot of Temperature sensor 30 cm underwater.

**Fig. 17:**
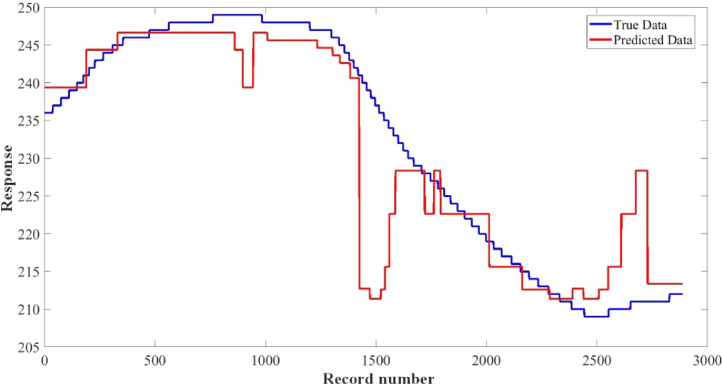
Regression method response plot of Turbidity sensor 30 cm underwater.

**Fig. 18:**
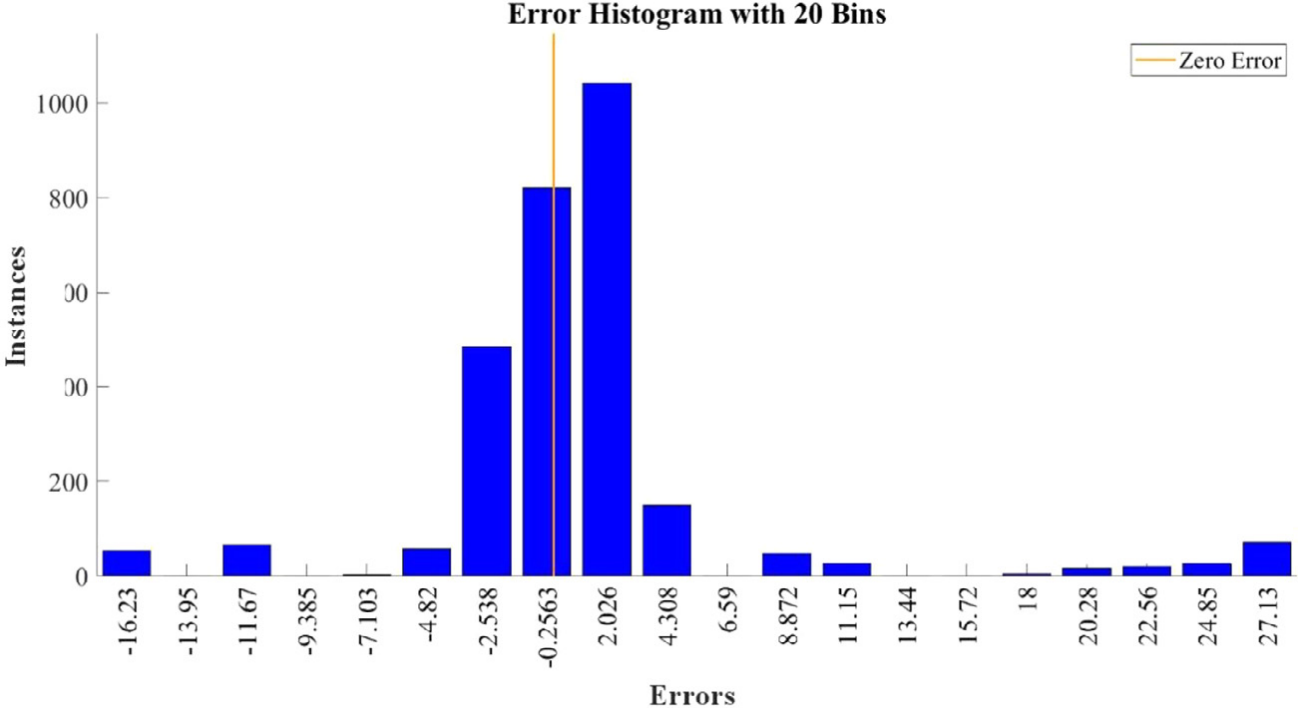
Error histogram plot of Turbidity sensor 30 cm underwater.

**Fig. 19:**
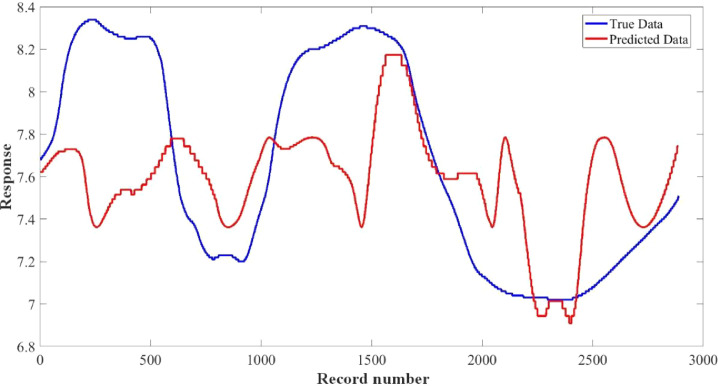
Regression method response plot of pH sensor 30 cm underwater.

**Fig. 20:**
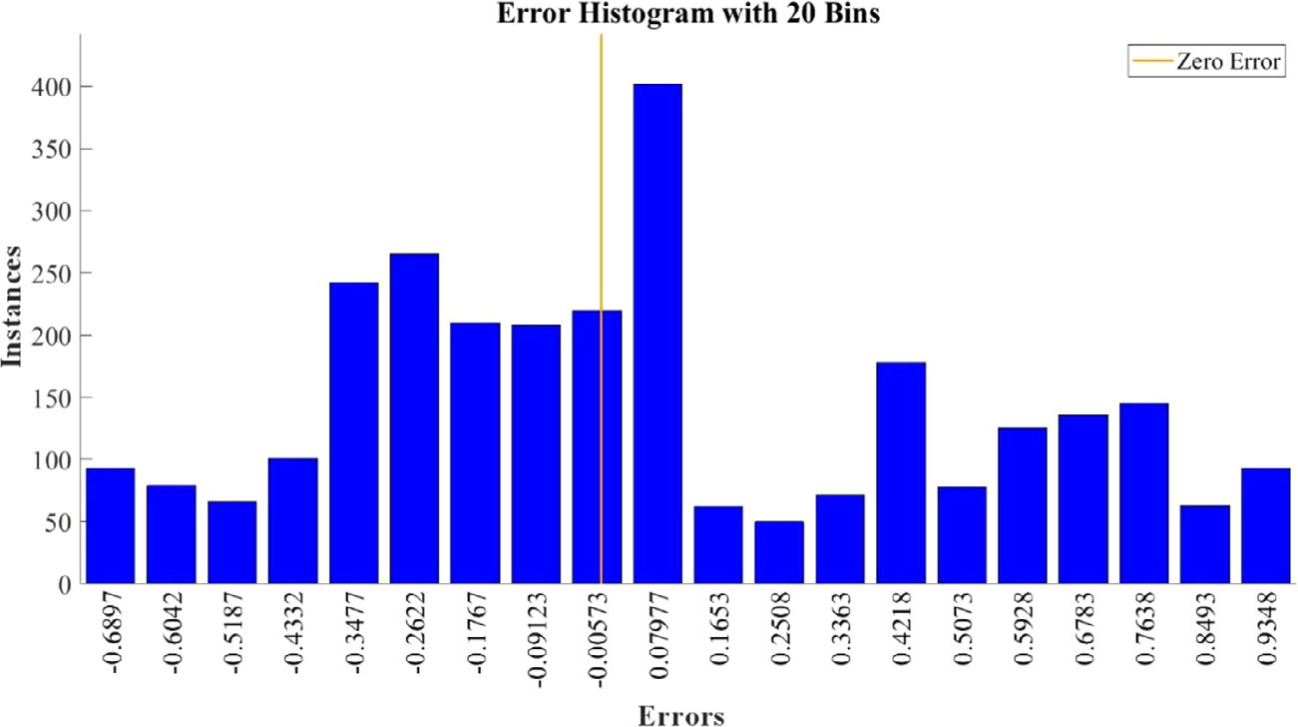
Error histogram plot of pH sensor 30 cm underwater.

**Fig. 21:**
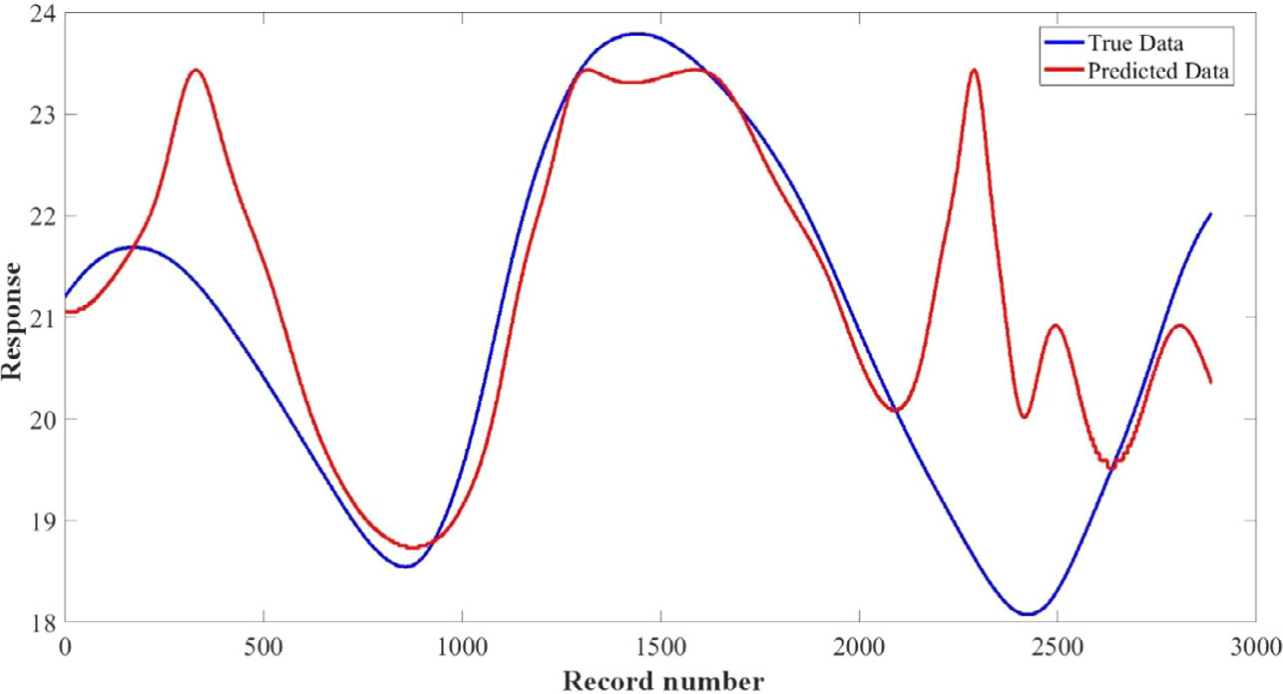
Regression method response plot of Temperature sensor 60 cm underwater.

**Fig. 22:**
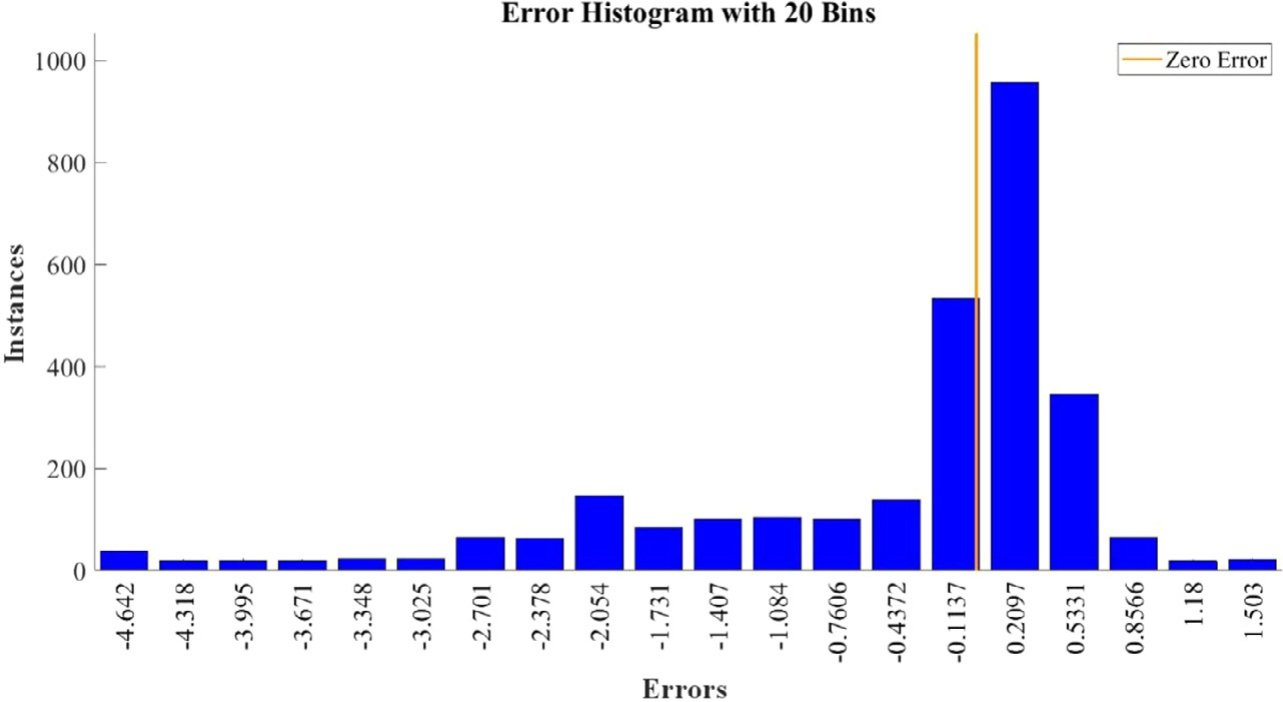
Error histogram plot of Temperature sensor 60 cm underwater.

**Fig. 23:**
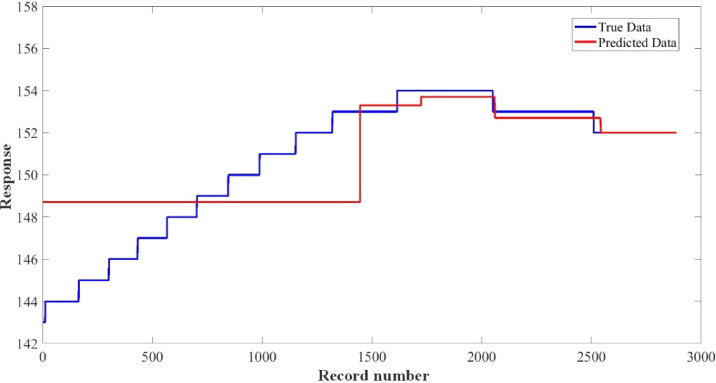
Regression method response plot of Turbidity sensor 60 cm underwater.

**Fig. 24:**
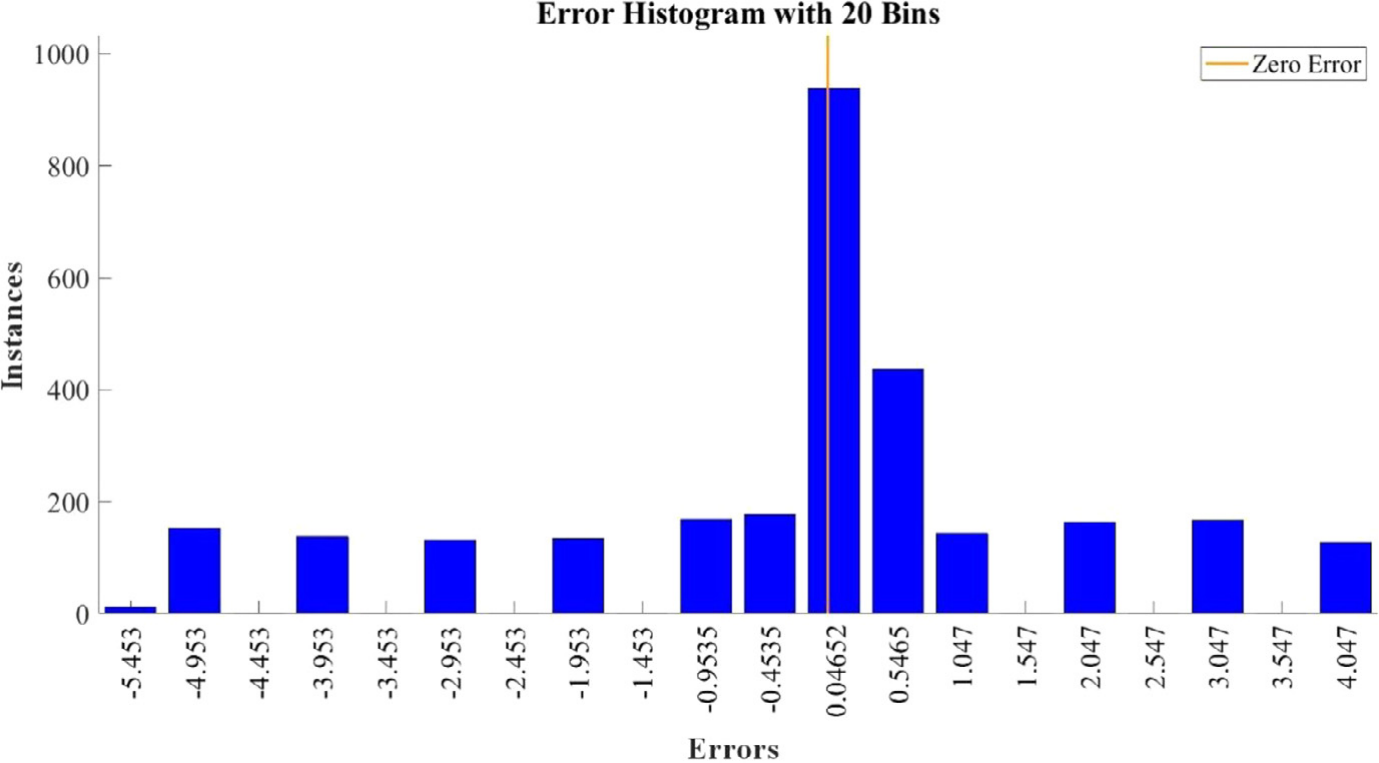
Error histogram plot of Turbidity sensor 60 cm underwater.

Figs. 15–24 show the response plots and the corresponding error histogram plots of machine learning regression method (Support Vector Machine) of the dataset. 70% data of the dataset from each sensor was used for training the regression model and the rest 30% was used for testing. Figs. 15, 17, 19, 21 and 23 show the true data vs. predicted data plots where blue line represents true data and red line represents predicted data. Figs. 16, 18, 20, 22 and 24 show the error histogram plots of the corresponding response plots.

## 2 Experimental Design, Materials and Methods

Before developing the system's algorithm identifying the most important water quality factors is necessary. Factors that affect water quality the most are needed to be monitored. Hence several quality factors were analyzed that have the maximum impact in the aquatic environment of a fish farm [3,4]. Based on that the sensors were selected for monitoring the respective parameters. Selected water quality factors and respective sensors are described below:

### 2.1 Hardware and sensors

#### 2.1.1 pH level and pH sensor

Water quality greatly depends on the pH factor, whether the water is acidic or nonacidic. Different fish like different kinds of pH conditions. So depending on which type of fish is being cultivated in the farm the pH factor can be observed to calculate the water suitability. Cellular membranes of a fish get damaged in high pH level like 9–14. Where low pH levels affects the rocks in the sediment resulting in release of metals. This increases water turbidity. Therefore a pH meter was used to collect the pH rating of the water.

#### 2.1.2 Temperature level and temperature sensor

Maximum fresh water fishes have cold blood. That means they collect temperature from their surrounding water, thus synchronizing with the water temperature. Cold blooded animals are affected directly by its surrounding medium temperature. Temperature mainly affects their metabolism [5]. As a result rapid change in water temperature causes the fish stress and may harm their growth. Therefore two waterproof Temperature sensors were used to measure the temperature of the pond.

#### 2.1.3 Turbidity level and turbidity sensor

If there is a lot of suspended material in the water or an excessive amount of food it may make the water dirty. Moreover high turbidity because of algae present in the water can harm fishes. Such as Trichodiniasis is a disease that happens due to parasites. Also turbidity affects the growth of fish eggs and larvae [6]. Furthermore light will not pass through a dirty water and organic materials may cause poisoning. For this reason two Turbidity sensors were used to measure the turbidity level of the water.

#### 2.1.4 Arduino mega and supporting modules

To maintain the sensors, collect and store the data an Arduino Mega microcontroller board was used in this project. This board has enough input and output pin and processing power to support all the modules perfectly. To store data in the cloud storage an ESP8266 Wi-Fi Module was used. And to store data in an electronic storage device a micro sd card reader module was used.

### 2.2 Methods

**Fig. 25:**
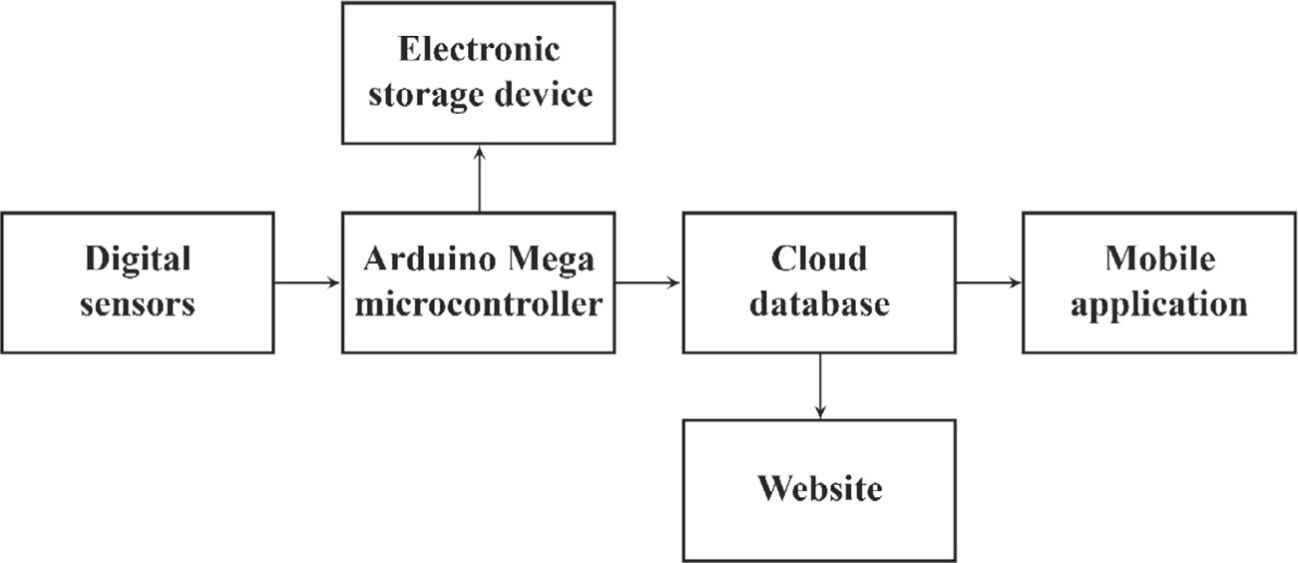
Block diagram of the data collection and storing system.

In the fish pond total number of deployed sensors were five divided into two sets. The first set includes a Temperature sensor, a pH sensor and a Turbidity sensor. This set of sensors were 30 cm underwater from the water surface. A second set of sensors were used in a different depth. Because the temperature and turbidity rating in water changes with respect to depth. The second set includes a Temperature sensor and a Turbidity sensor. This set was 60 cm underwater from the water surface. Both sets had the same horizontal alignment yet different depth. The sensors were immersed in the water where the microcontroller and the other modules were above the water surface attached to a floating structure. The microcontroller board read data from the sensors all together. Then uploaded the data in the cloud database and stored in the storage device. Rate of data record on average was one set of data per minute. Later data was presented in a dedicated website for monitoring them from anywhere. For portable monitoring a mobile application had been developed. The website and the mobile application shows real time conditions of the water at any time. The mobile application is capable of notifying the user when any one of the parameters of the water quality factor crosses the safety limit. The overall data collection and storing system is presented in Fig. 25 and briefly described in Algorithm I.

Algorithm I: Algorithm for collecting and storing the sensor data

• Step 1: Delay 30 seconds for calibration of the sensors.
• Step 2: Read data *X* and *Y* from the Temperature sensors.
• Step 3: Read data *Z* from the pH sensor.
• Step 4: Read data *P* and *Q* from the Turbidity sensors.
• Step 5: Write the data *X, Y, P, Q* and *Z* in the electronic storage device.
• Step 6: Delay 200 ms.
• Step 7: Try to establish connection with the web host.
• Step 8: If connection is established go to Step 9, else go back to step 7.
• Step 9: Upload data in the database.
• Step 10: Delay 300 ms.
• Step 11: Go back to Step 2.

**Fig. 26:**
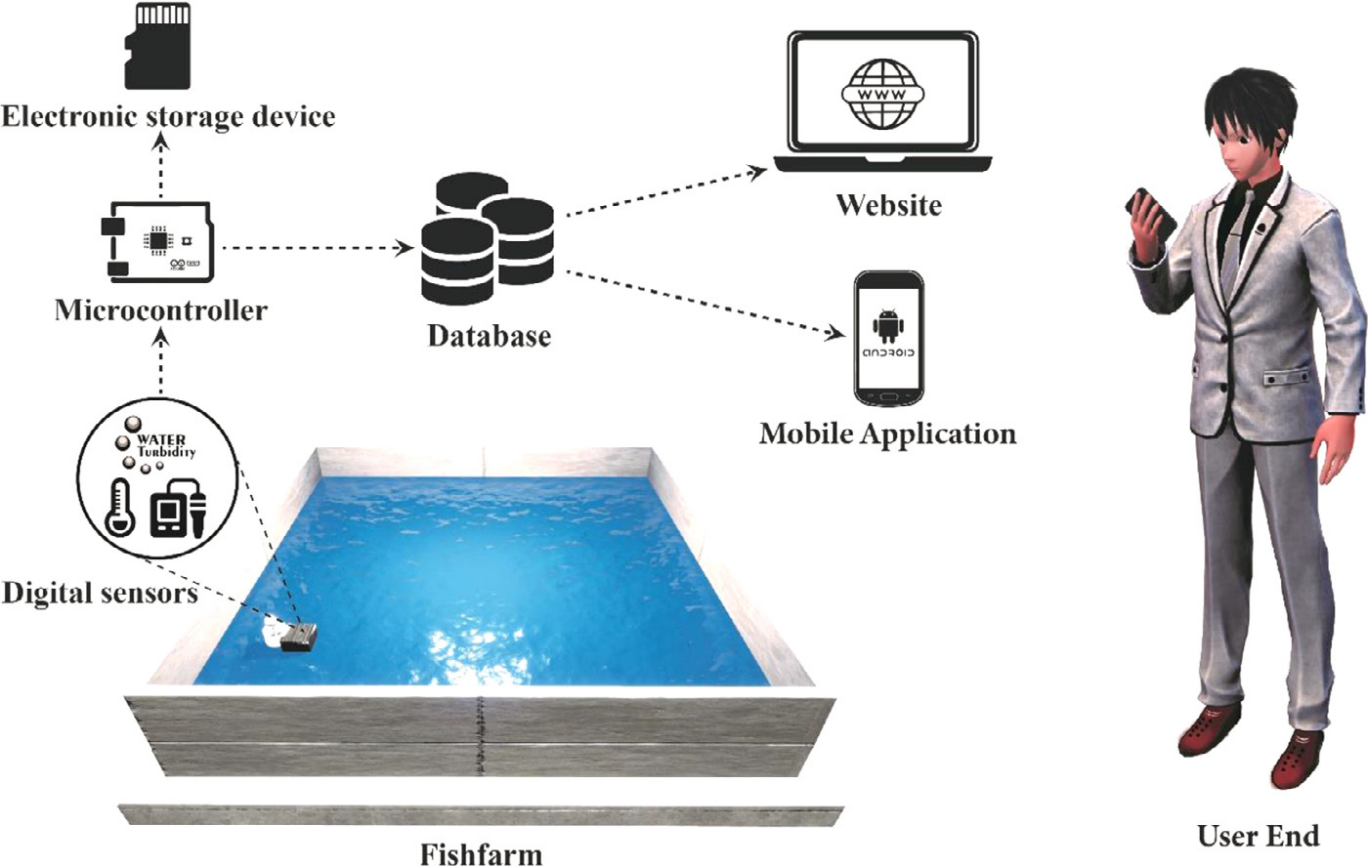
Schemetic diagram of the overall data collection and monitoring system.

Fig. 26 illustrates the overall data collection and monitoring system. It shows how data were collected, stored and finally presented to the user.

### 2.3 Data collection and presentation

**Fig. 27:**
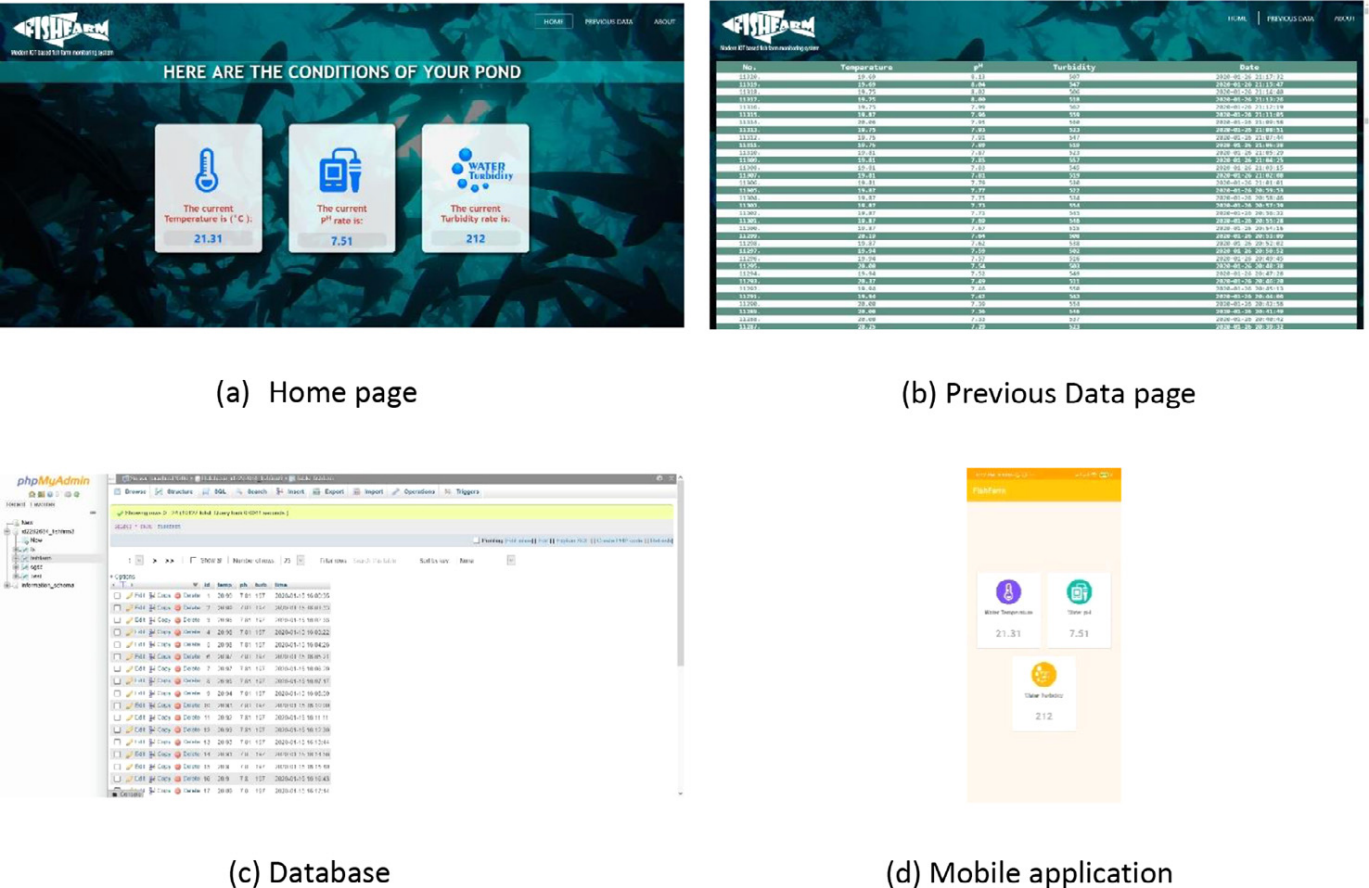
Website layout, Database and Mobile application.

Data were presented in a dedicated website for monitoring the farm condition from anywhere and anytime. The cloud data collection and presentation system is shown in Fig. 27. The ‘Home page’ of the website let a user monitor each parameter individually in real time. It updates with new data in every minute. Fig. 27a shows the layout of the ‘Home page’ of the website. The previous data of the parameters can be found in the ‘Previous Data’ page. This page provides all the previous data in a descriptive manner. Fig. 27b shows the layout of the ‘Previous Data’ page of the website. Data were collected both from the database and the storage device so that no data were missed. Fig. 27c shows the database of the work. A mobile application also supported in collecting data and made necessary notifications. Fig. 27d shows the layout of the mobile application.

### 2.4 Machine learning and prediction

Machine learning regression method was used to predict near future data and compare them with the true data. For this purpose we used the Gaussian kernel of Support Vector Machine (SVM) learning model. 70% of the data was used to train the regression model and 30% was used to test it. Later prediction error was calculated by subtracting the predicted data from the true data.

Matlab's script command “fitrsvm(x,y)” was used to train and test the SVM regression model. The script functions used for fitting is given below:

**Table:** 

mdl = fitrsvm(x,y,'KernelFunction','gaussian');	%Training the model with predictor x and response y
ypred = resubPredict(mdl);	%Predicting data based on the trained model
e = y-ypred;	%Calculating error

### 2.5 Arduino sketch

#### 2.5.1 Reading data from the sensors

**Fig. 28:**
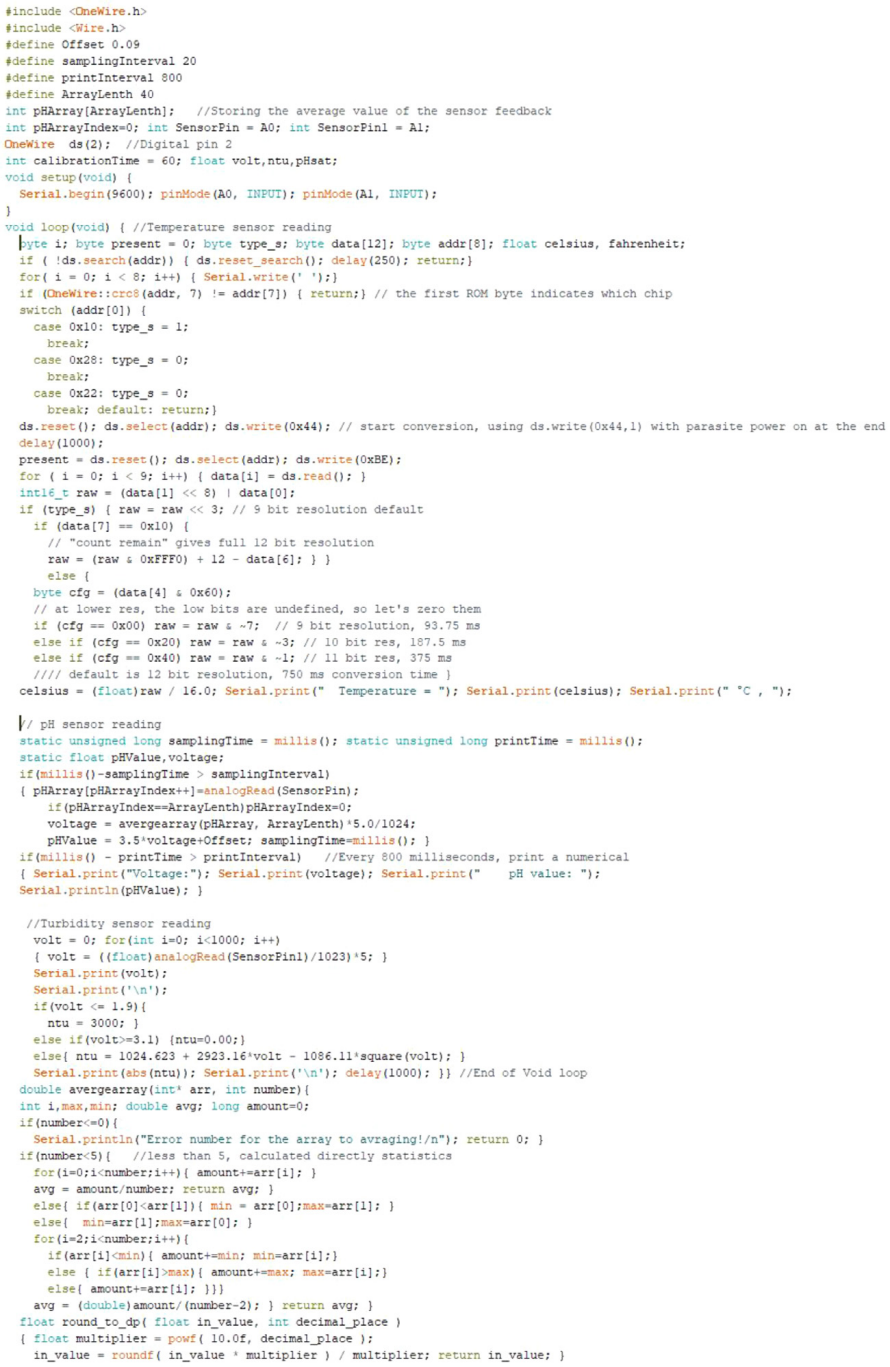
Arduino sketch for reading different sensors.

Fig. 28 shows the Arduino sketch for reading data from the three types of sensor used in this work.

## Ethics Statement

Not applicable.

## Declaration of Competing Interest

The authors declare that they have no known competing financial interests or personal relationships which have, or could be perceived to have, influenced the work reported in this article.
